# CT and MR Appearance of Teeth: Analysis of Anatomy and Embryology and Implications for Disease

**DOI:** 10.3390/jcm13051187

**Published:** 2024-02-20

**Authors:** Zachary Abramson, Chris Oh, Martha Wells, Asim F. Choudhri, Matthew T. Whitehead

**Affiliations:** 1 Clinical Radiology, Radiologist, Body Imaging, Department of Diagnostic Imaging, St. Jude Children’s Research Hospital, 262 Danny Thomas Place, Memphis, TN 38105, USA; 2 Quantum Radiology, 790 Church St., Suite 400, Marietta, GA 30060, USA; coh@quantumrad.com; 3 Department of Surgery, St. Jude Children’s Research Hospital, Memphis, TN 38105, USA; martha.wells@stjude.org; 4 Department of Radiology, Le Bonheur Children’s Hospital, University of Tennessee Health Science Center, 50 N. Dunlap St., Memphis, TN 38103, USA; achoudhri@uthsc.edu; 5 Department of Radiology, Perelman School of Medicine, University of Pennsylvania, Philadelphia, PA 19104, USA; whiteheadm@chop.edu; 6 Division of Neuroradiology, Children’s Hospital of Philadelphia, Philadelphia, PA 19104, USA

**Keywords:** teeth, embryology, anatomy, computed tomography, magnetic resonance imaging, odontogenic infection

## Abstract

Abnormalities of dental development and anatomy may suggest the presence of congenital or acquired anomalies. The detection of abnormalities, therefore, is an important skill for radiologists to achieve. Knowledge of dental embryology and an understanding of the radiologic appearances of teeth at various stages of maturation are required for the appreciation of abnormal dental development. While many tooth abnormalities are well-depicted on dedicated dental radiographs, the first encounter with a dental anomaly may be by a radiologist on a computed tomographic (CT) or magnetic resonance (MR) exam performed for other reasons. This article depicts normal dental anatomy and development, describing the appearance of the neonatal dentition on CT and MRI, the modalities most often encountered by clinical radiologists. The radiology and dental literature are reviewed, and key concepts are illustrated with supplemental cases from our institution. The value of knowledge of dental development is investigated using the analysis of consecutive MR brain examinations. Finally, the anatomical principles are applied to the diagnosis of odontogenic infection on CT. Through analysis of the literature and case data, the contrast of dental pathology with normal anatomy and development facilitates the detection and characterization of both congenital and acquired dental disease.

## 1. Introduction

Abnormalities of dental development and anatomy often suggest the presence of congenital or acquired anomalies [1,2]. The detection of abnormal dental development or anatomy, therefore, is an important skill for radiologists to achieve. Knowledge of dental embryology and an understanding of the radiologic appearances of teeth at various stages of maturation are required for the appreciation of abnormal dental development and anatomy.

While a complete description of the radiologic appearance of teeth at all stages of development from fetal to and throughout adult life is ideal, investigating the neonatal dentition is valuable for several reasons: (1) the variable stages of dental development present in the neonatal period represent most of the developmental stages seen throughout life; (2) these stages also include cellular and mineralized tissues, providing a wide range of imaging appearances helping to demonstrate key radiology principles; (3) abnormalities that appear at this age are more likely associated with other anomalies; (4) understanding early dental development, as seen in the neonatal period, reinforces the appreciation for mature tooth anatomy, which is needed to identify acquired conditions, such as infection.

While many abnormalities of the dentition are well-depicted on dedicated dental radiographs [3], the first encounter with a dental anomaly may be by a radiologist on a computed tomographic (CT) or magnetic resonance (MR) exam performed for other reasons [4,5,6]. Further, most dental radiographs are interpreted by dental clinicians and not viewed by a radiologist in a medical setting. This review describes normal dental anatomy and development, describing the appearance of the neonatal dentition on CT and MRI, the modalities most-often encountered by clinical radiologists. The radiology and dental literature are reviewed, and key concepts are illustrated with supplemental cases from our institution. We analyze a consecutive series of neonatal brain MR examinations demonstrating the clinical value of knowledge of dental development. Finally, anatomical principles are applied to the diagnosis of odontogenic infection on CT. The focus on imaging modalities more commonly encountered by non-dental specialists is intended to broaden the knowledgebase of providers who may see the patient or patient images prior to referral to a dentist. The information presented in this manuscript provides a foundation for understanding dental imaging at all stages of dental development.

## 2. Materials and Methods

A combination of journal articles, textbooks and personal educational materials by the authors were used to summarize the anatomy and embryology of developing teeth and their radiologic appearances on CT and MRI. The knowledge and principles learned through this investigation were then applied to a series of patients who underwent neonatal brain MR imaging for a variety of reasons. Consecutive-term neonatal patients who underwent brain MR at our institution were retrospectively analyzed after IRB approval. The review was performed by two neuroradiologists with American Board of Radiology (ABR) subspecialty certificates in neuroradiology with clinical practices focusing on pediatric neuroradiology. Demographic, clinical, and radiological data were compiled in tabular form and descriptive statistics were computed. The application of dental anatomical principles to the diagnosis of odontogenic infection was shown using case examples.

## 3. Results

### 3.1. Anatomy of the Dental Arch

**Figure 1:**
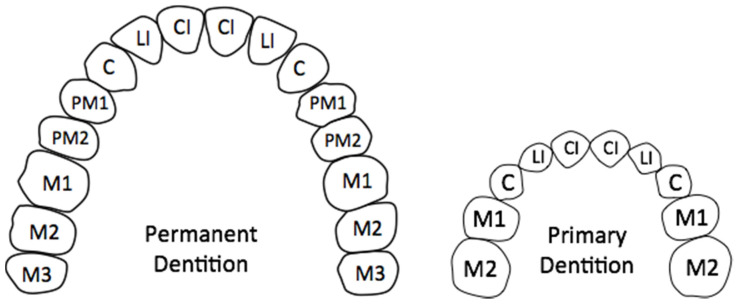
Arrangement of teeth within permanent and primary dental arches.

In adult humans, a full dental complement comprises 32 teeth, housed in 2 bilaterally symmetric arches [7]. In each quadrant (half an arch) beginning in the midline, humans have two incisors (central and lateral), one canine, two premolars (first and second), and three molars (first, second, and third) (Figure 1). Similar to the adult dentition, the primary dentition is housed in two bilaterally symmetric arches. In a full complement, each quadrant contains (beginning from midline) two incisors (central and lateral), one canine, and two molars (first and second) (Figure 1). By convention, the direction along the arch toward the midline is referred to as mesial, whereas the direction on the arch away from the midline is termed distal.

**Figure 2:**
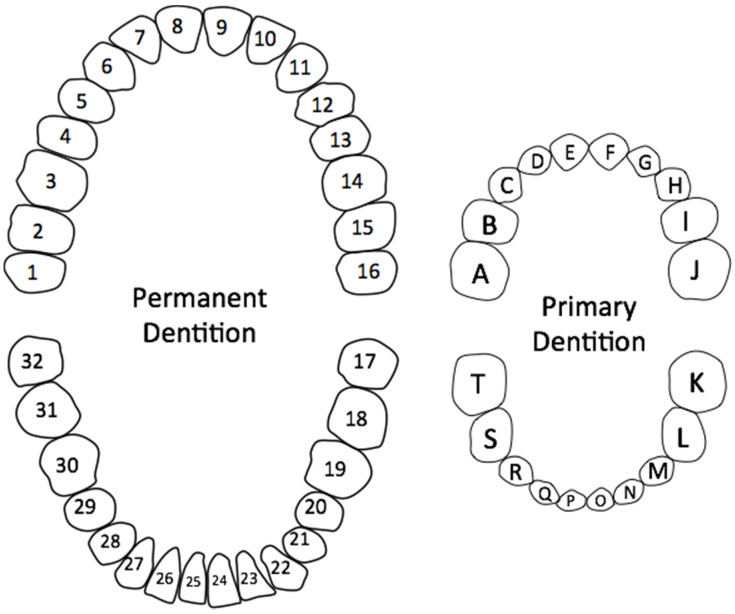
American Dental Association Universal Numbering/Lettering System for permanent and primary dentition.

Several different dental notation systems exist, and not all dental professionals use the same system. Consequently, the best way to refer to teeth mimics the following paradigm: side; arch; tooth (i.e., right maxillary lateral incisor)—in particular when referring to primary dentition. Familiarity with the common numbering system used for adult teeth is also important (Figure 2) [8].

**Figure 3:**
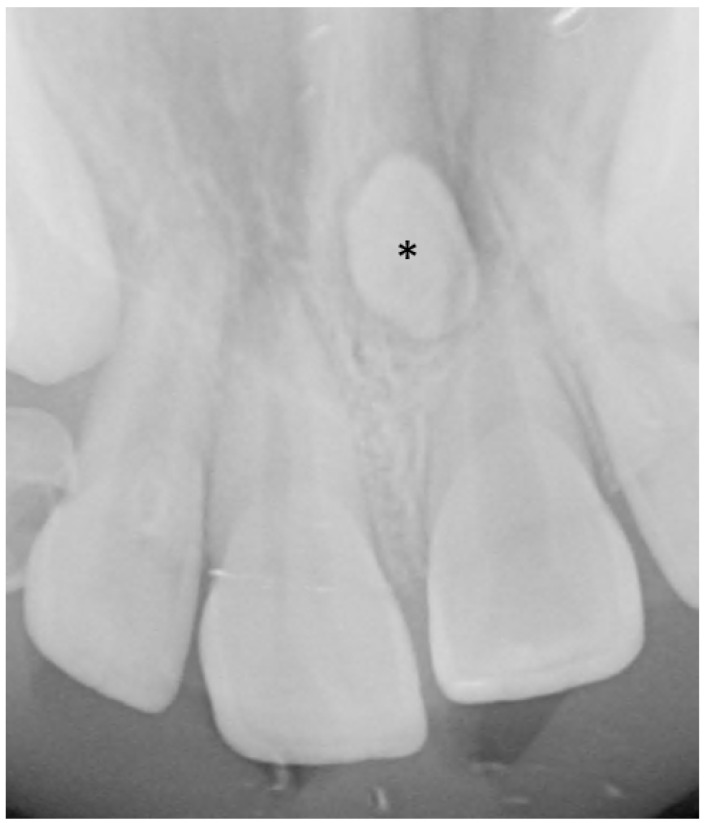
Supernumerary tooth (*) situated in between the roots of the central incisors, termed a mesiodens, shown on a dedicated dental radiograph.

**Figure 4:**
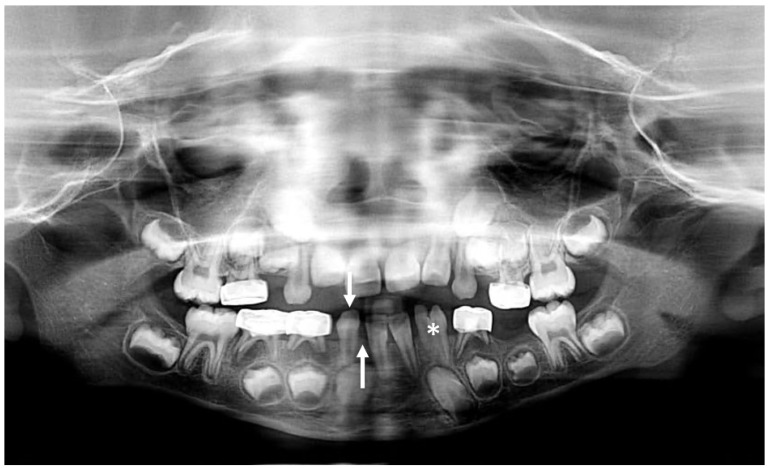
Panoramic radiograph demonstrating congenital absence of right mandibular lateral incisor (up arrow), manifesting as increased space surrounding the right mandibular canine (down arrow). The left mandibular lateral incisor and canine are fused (*). When counted as one tooth, the patient had one less than expected tooth in the lower left quadrant, confirming that this was a fusion of two teeth as opposed to the gemination of a single tooth.

**Figure 5:**
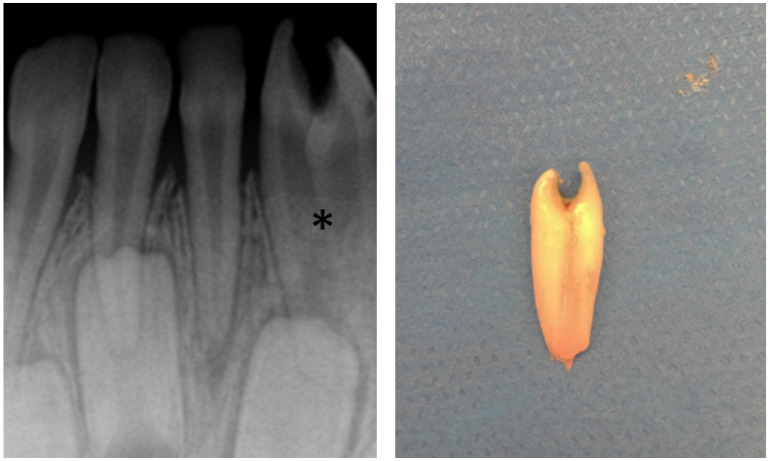
Geminated primary lower lateral incisor shown radiographically (*) and photographically following extraction. When counted as one tooth, the patient had the appropriate number of teeth, confirming this was a geminated tooth as opposed to the fusion of two teeth.

An abnormal number of teeth can result from missing or supernumerary teeth. The most common supernumerary tooth is located between the two central incisors, termed a mesiodens (Figure 3) [9]. Excluding third molars, the most common congenitally missing permanent teeth are maxillary lateral incisors and mandibular premolars followed by maxillary lateral incisors and maxillary second premolars [9,10]. Congenital absence of primary teeth is very rare and is generally, but not always, followed by agenesis of the succedaneous tooth [11,12]. The general term for less than a full complement of teeth is hypodontia, whereas the term for extra teeth is hyperdontia. Since third molar development is variable, missing third molars is not typically categorized as hypodontia. When few teeth develop, the term ‘oligodontia’ can be used. Two other processes can lead to confusion in the number of teeth present: fusion and gemination. Fusion occurs when two developing tooth germs partially merge during development (Figure 4). Gemination of a tooth germ results in partial twinning of a tooth germ during development (Figure 5). A useful tip for distinguishing between the two entities is to count the teeth: when a fused or geminated tooth germ is present and counted as one tooth, the total number of teeth will be one shy of the normal complement in the setting of fusion, but normal in gemination [13].

#### 3.1.1. Anatomy of Tooth Structure

**Figure 6:**
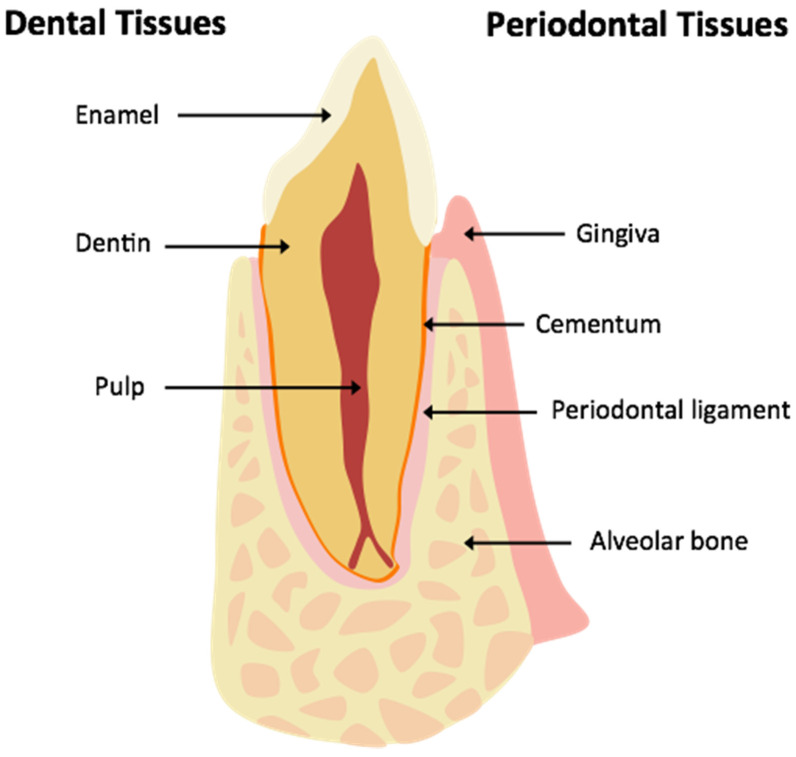
Basic dental and periodontal anatomy.

The mature tooth is composed of enamel, dentin, and pulp [14]. The tooth can be divided into two segments: the crown and root. The crown contains all three types of tissues whereas the root contains only dentin and pulp (Figure 6). The outer enamel layer of the crown is 96% inorganic and entirely acellular. Within the crown, just underneath the enamel lies the less mineralized dentin (70% inorganic). This layer is also acellular but contains cellular processes from the dentin-producing odontoblasts, which reside in the periphery of the underlying pulp. The pulpal tissue contains blood vessels, connective and lymph tissue, and nociceptive fibers, and has a coronal component (pulp chamber) as well as a root component (pulp canal) [15]. The tip of the root, where nerves and blood vessels enter the pulp, is termed the apex [16]. It is an important structure as it is the last structure to be formed and failure to form a narrow apex may suggest pathology.

#### 3.1.2. Periodontal Tissues

**Figure 7:**
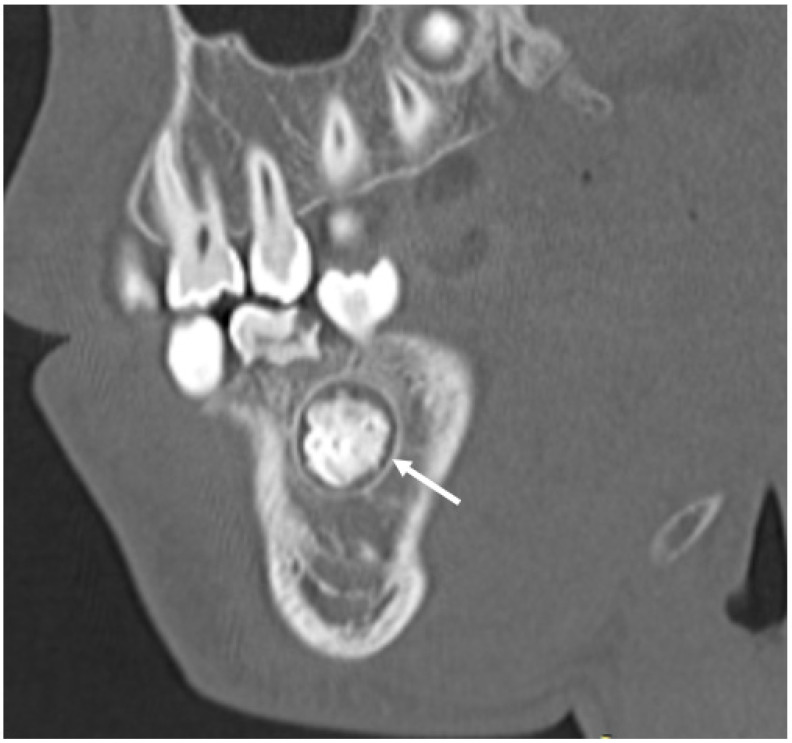
Sagittal section CT demonstrating heterogeneous mineralized tissues with evidence of bone and enamel densities. The uniform surrounding radiolucency (white arrow) suggests the presence of a periodontal ligament space compatible with a diagnosis of an odontoma.

The tissues surrounding the tooth, termed the periodontium, consist of cementum, periodontal ligament, alveolar bone, and gingiva. Cementum is the thin cellular layer surrounding the root, which allows attachment of the periodontal ligament. The periodontal ligament is a thin (200 microns) tissue layer that extends from the alveolar bone to the cementum [17]. The presence of this periodontal ligament space around a focus of very dense but disorganized tissues may suggest odontoma (Figure 7). Alveolar bone houses and supports the teeth within the maxilla and mandible. Finally, the gingiva is the soft tissue covering of the alveolar bone and forms an important tooth–mucosa junction (Figure 6).

### 3.2. Dental Development

#### 3.2.1. Initiation of Odontogenesis and Bud Stage

Odontogenesis results from a complex interaction between the specialized oral epithelium termed the dental lamina and the underlying mesenchyme derived from neural crest cells, termed ectomesenchyme [18,19,20]. It is the mesenchymal cells that first signal the initiation of tooth formation, through a process termed induction. In response to inductive signals, portions of the dental lamina proliferate downwards into the mesenchyme, forming a tuft of epithelial cells with surrounding mesenchyme called a tooth bud or bud stage [21].

#### 3.2.2. Cap Stage

Secondary to unequal epithelial proliferation, the tuft of cells begins to form a cap around densely packed mesenchymal cells. This stage, referred to as the cap stage, marks the beginning of morphodifferentiation and histodifferentiation. A depression forms in the cap of epithelial cells, creating the enamel organ, which will produce the future enamel. The condensing mass of mesenchyme is now referred to as the dental papilla, which will form the future dentin and pulp tissue. The surrounding sac, also composed of mesenchyme, forms the dental follicle, which eventually gives rise to the periodontal tissues [21]. The enamel organ, dental papilla, and dental follicle comprise the tooth germ. A tooth germ may be referred to as a tooth bud, but this should not be confused with the bud stage (Table 1).

#### 3.2.3. Bell Stage

**Figure 8:**
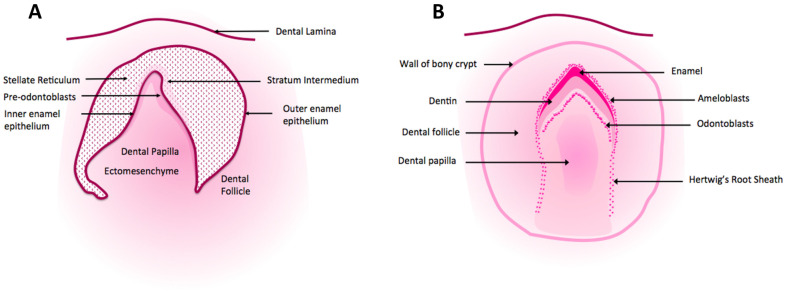
Diagram representation of histology of tooth germ in the bell stage (**A**) and post-apposition maturation stage (**B**). The differentiating features include the collapse of the dental organ and presence of mineralized tissue in the bell stage. The transition between these stages has corresponding imaging findings (see radiological correlations section).

As histodifferentiation and morphodifferentiation continue, the cap assumes a bell shape. The enamel organ contains an inner and outer enamel epithelium and central cellular substances termed stellate reticulum and stratum intermedium, which serve as nutritional support to the enamel epithelium, allowing the enamel organ to separate from the dental lamina. The cells of the stellate reticulum produce glycosaminoglycans, which draw water into the enamel organ, providing further structural support to the enamel organ and contributing to folds to create the appropriate shape of the future crown. Additionally, during this stage, the dental papilla separates into an outer layer of future dentin-secreting cells (odontoblasts) and a central mass of cells that forms the primordium of the future pulp. The follicle at this time is mostly amorphous and increases collagen production (Figure 8A) [21]. 

It is at about this time that the primordia for the permanent teeth may arise as extensions of the epithelium associated with the primary tooth germ. Permanent teeth that arise in this fashion are referred to as succedaneous teeth. Non-succedaneous teeth include all permanent molars and are thought to arise separately from posterior extensions of the dental lamina.

#### 3.2.4. Apposition and Maturation

At the end of the bell stage, there is a collapse of the dental organ with loss of the glycosaminoglycan layer and consequent loss of water. This collapse allows for simple diffusion to provide nutrients to the inner enamel epithelium, which is now terminally differentiated. The cells of the inner enamel epithelium, now termed ameloblasts, and the outer cells of the dental papilla, now termed odontoblasts, are primed to secrete the precursors to enamel and dentin, respectively. This next phase is known as the appositional stage, where partially calcified enamel and dentin matrices are secreted and serve as a framework for further calcification. The maturation stage is characterized by completion of calcification (Figure 8B).

#### 3.2.5. Root Formation

As the crown continues calcifying, the roots are still developing. At the edges of the crown, the inner and outer enamel epithelium proliferates downwards, forming a double-layered sheath separating the dental papilla from the surrounding follicle, termed Hertwig’s root sheath. Unlike in the enamel organ, there is no stellate reticulum or stratum intermedium in the root sheath and, therefore, no ameloblasts are formed. The odontoblasts of the outer dental papilla interact with the root sheath and are signaled to produce the dentin of the root. The basement membrane of the root sheath subsequently breaks down, and specialized cells of the dental follicle termed cementoblasts produce cementum along the newly exposed dentin surface. While the cementum is forming on the roots, the central cells of the dental papilla form the pulp chamber and canal system. Other specialized cells in the dental follicle go on to form the periodontal ligament attaching to cementum and adjacent alveolar bone [21]. This process continues until the sheath converges at the root apex, which may 2–3 years after the crown of the tooth erupts into the oral cavity [22,23,24].

#### 3.2.6. Eruption

Eruption sequences are fairly consistent and well-documented in the literature but are beyond the scope of this article [25,26]. All primary tooth germs and first permanent molar tooth germs are detectable by MRI at birth [27]. It is important to note, however, that at birth, none of the teeth are erupted into the oral cavity. An erupted tooth at birth is termed a natal tooth, and a tooth which erupts within the first 30 days of life is termed a neonatal tooth, both of which are abnormal but may or may not be associated with pathology [28,29]. The majority of natal and neonatal teeth (≥90%) are the primary teeth (most commonly the mandibular incisors) and not supernumerary teeth [30]. Another important pearl is that of symmetry. Development tends to be bilaterally symmetric and, to a lesser degree, symmetric among the maxillary and mandibular arches. When correlating cross-sectional findings with radiographs, it should be noted that significant mineralization is required for a developing tooth to appear opaque on X-ray, whereas the unmineralized tooth germ can be visualized much earlier on CT or MRI, as further detailed below.

#### 3.2.7. Post-Maturation Changes

**Figure 9:**
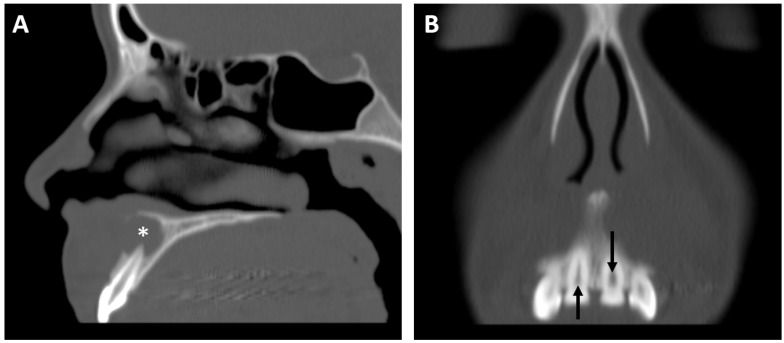
An 18-year-old female with tooth pain. (**A**) Sagittal-reformatted CT shown in bone windows reveals a periapical lucency (asterisk) involving the left maxillary central incisor, compatible with a cyst, granuloma, or abscess, in keeping with a diagnosis of periapical periodontitis. (**B**) Coronal-reformatted CT demonstrating a widened pulp chamber of the left maxillary central incisor (down arrow) relative to the right maxillary central incisor (up arrow), indicative of remote/chronic pulpal necrosis.

Once a tooth is fully formed, the odontoblasts continue to lay down new dentin, which gradually narrows the pulp chamber and canal. The cessation of continued dentin formation and narrowing of the pulp chamber is a radiologic sign of pulpal necrosis (Figure 9).

### 3.3. Radiologic Correlations of Embryological Development

**Figure 10:**
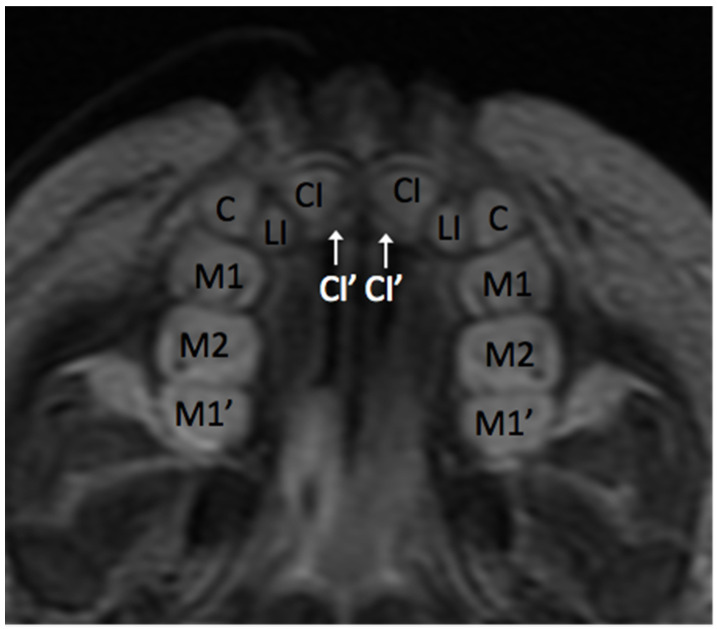
Axial section T2WI MRI: primary maxillary teeth in maturation stage with permanent maxillary central incisors (CI′) and first molars (M1′) in pre-appositional stages.

**Figure 11:**
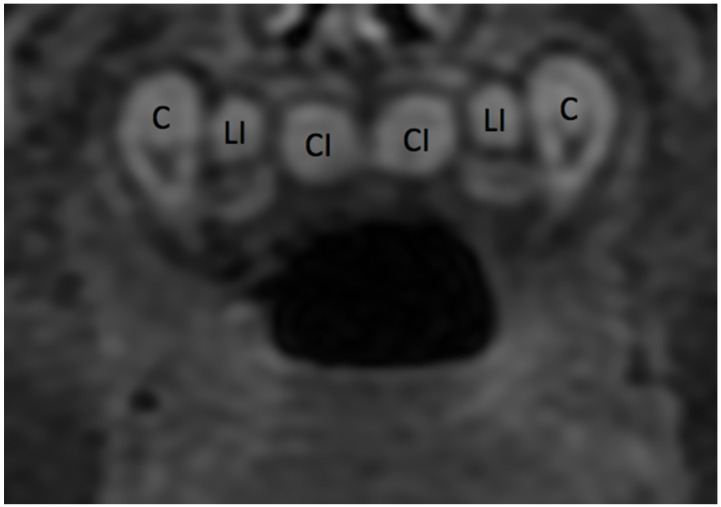
Coronal section T2WI MRI: primary maxillary canines and incisors in maturation stage of development.

**Figure 12:**
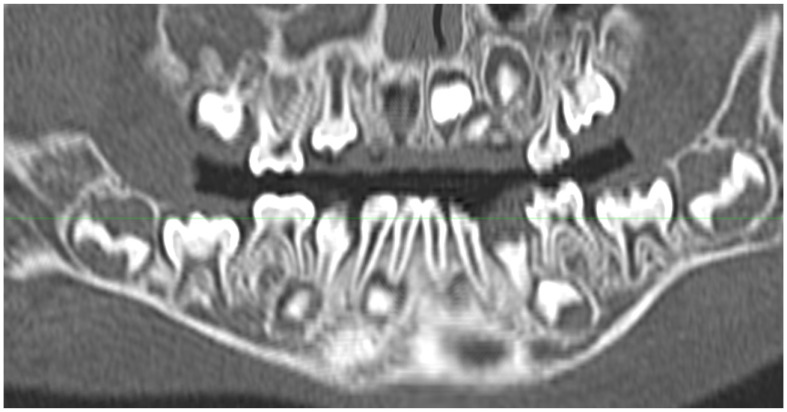
Curved plane coronal CT reconstruction in a 28-month-old child, presented in a manner similar to that of panoramic tomography. The horizontal green line reflects an image processing artifact.

**Figure 13:**
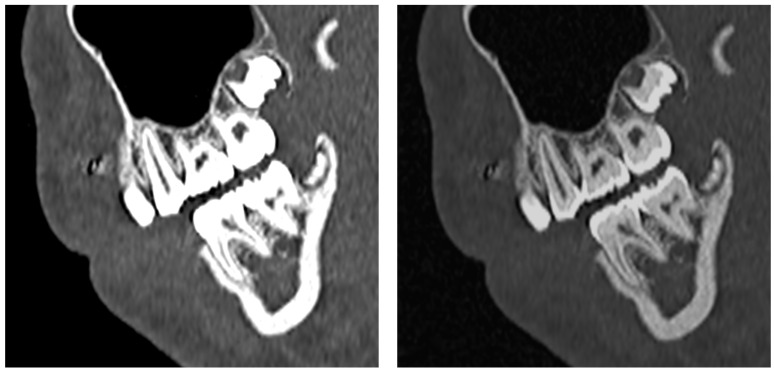
Sagittal section CT shown in bone window and level (**left**). On the **right**, the window has been widened and the level has been increased to allow demonstration of the enamel and dentin components of the dentition.

**Figure 14:**
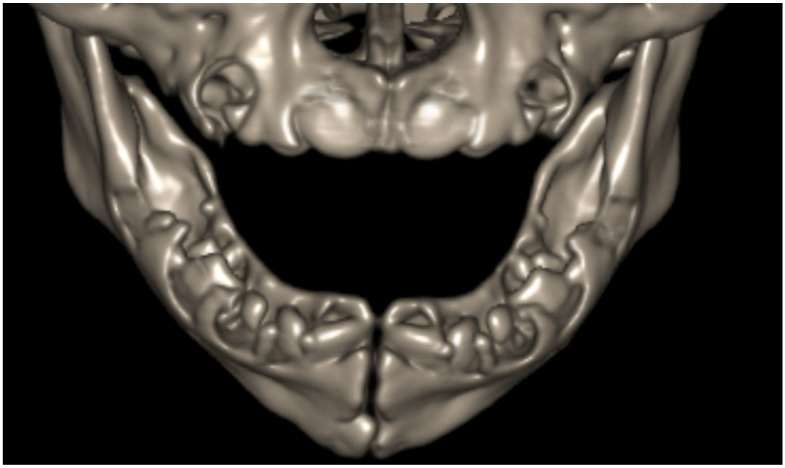
Three-dimensional CT reconstruction of the dental arches in a neonate revealing the numerous bony crypts in which multiple primary teeth are developing.

Dental arches are readily identified on imaging by the presence of teeth or tooth germs. On axial imaging, the dental arch is U-shaped. Axial sections demonstrate arch asymmetry nicely and can be viewed to quickly screen for missing or supernumerary teeth. Axial imaging also depicts the presence of both primary and secondary teeth on the same image, with secondary teeth arising lingual to the primary dentition (Figure 10). Coronal and sagittal imaging can further detect dental abnormalities, including displacement and associated lesions (Figure 11). Curved-plane coronal CT reformats can be performed to show the entirety of the dental arches in a manner similar to that of panoramic tomography (Figure 12) [31,32]. When viewing the cross-sectional anatomy of teeth using CT, it is critical to appropriately set the window and level of the image to be able to distinguish the enamel and dentin layers. Wider window levels increase visibility of the dentin–enamel junction (Figure 13) [33]. Three-dimensional CT reconstructions can also be performed of the dental arches (Figure 14) [34,35,36]. Developmental abnormalities, such as agenesis and supernumerary teeth, can be determined simply by identifying the dental arches and counting teeth and teeth germs. More subtle abnormalities, however, require greater knowledge of the radiologic appearances of the teeth and teeth germs at various stages of development.

Visualization of subtle abnormalities can also be hindered by the limitations of imaging modalities. While modern CT scanners acquired images at sub-1 mm resolution, they are often reconstructed at thicker sections (~3 mm) to reduce image noise [37]. MRI, on the other hand, is typically acquired in thick sections with gaps between slices to reduce noise and limit the time on the MR scanner and/or under sedation. Due to these limitations, some abnormalities may be better demonstrated on conventional radiography, which exhibits excellent spatial resolution but suffers from the overlapping of structures inherent to a projection imaging technique in comparison to the cross-sectional techniques emphasized here.

There are a few radiologic principles that aid interpretation of dental development on MRI and CT:

1. Hydrophilic tissues have greater water content and consequent higher signal on T2-weighted sequences and decreased density on CT;
2. Calcification on MRI presents as hypointense on T2-weighted imaging. On CT, calcification presents as increased density with density greater or equal to bone.

**Figure 15:**
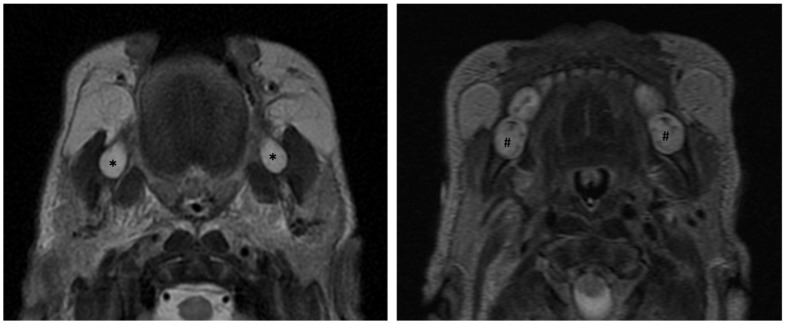
Axial section MRI demonstrating pre-appositional permanent mandibular first molars presenting uniformly hyperintense (*) and post-appositional primary second molars in the same patient presenting with areas of hypointensity/low signal (**#**).

**Figure 16:**
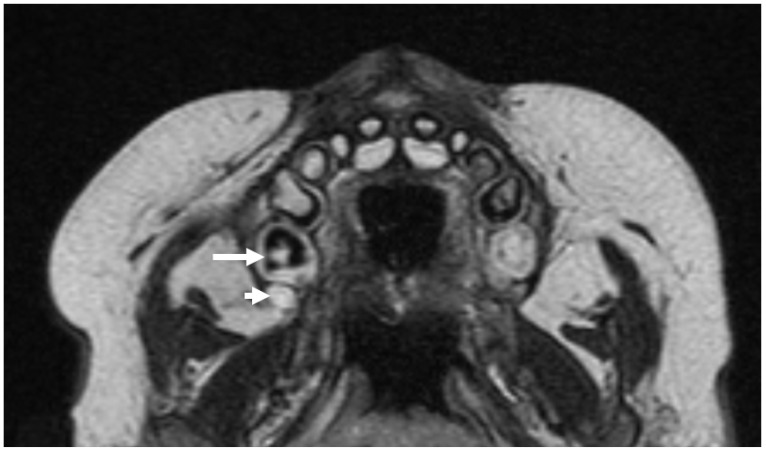
T2-weighted axial MRI through the maxillary dental arch/palate showing a developing primary second molar in the post-appositional phase (long arrow). Note the dark signal corresponding to areas of increased mineralization. Posterior to this tooth germ is a developing first permanent molar tooth germ in the earlier pre-appositional phase without internal areas of dark signal (short arrow).

The two simple principles stated above, when combined with the knowledge of dental anatomy and development allow for radiologic–anatomic correlations. Similar to brain imaging, the MR appearance of teeth varies with the stage of development. Analogous to the detection of abnormal myelination on MR, abnormal dental maturation can be readily assessed based on intrinsic T2 properties of developing teeth. Prior to the apposition of enamel and dentin, the developing tooth is largely hydrophilic, which leads to a hyper-intense signal on T2-weighted imaging. Following collapse of the enamel organ and apposition of enamel and dentin, the high mineral content largely devoid of water yields the hypo-intense regions on T2-weighted MRI (Figure 15 and Figure 16).

Identification of individual teeth can be aided by the cross-sectional appearance of the developing crown. Incisor crowns, which are described as wedge- or chisel-shaped, are seen as rectangles in the coronal plane and triangular in the sagittal plane. Canine crowns, on the other hand, are pyramidal in shape and are seen as triangular in coronal, sagittal, and axial sections. Molars and premolars, which are box-shaped, are rectangular in cross-sections. Keep in mind that displacements, rotations and abnormal eruption patterns can alter the appearance of crowns on cross-sectional imaging. When this occurs, 3D imaging or curved plane reformats can be employed. Molar crowns can be further identified by the presence of multiple peaks, called cusps. Pre-molars have two cusps, one buccal and one lingual. Molars have between four and five cusps with a central groove running in the mesial–distal direction.

**Figure 17:**
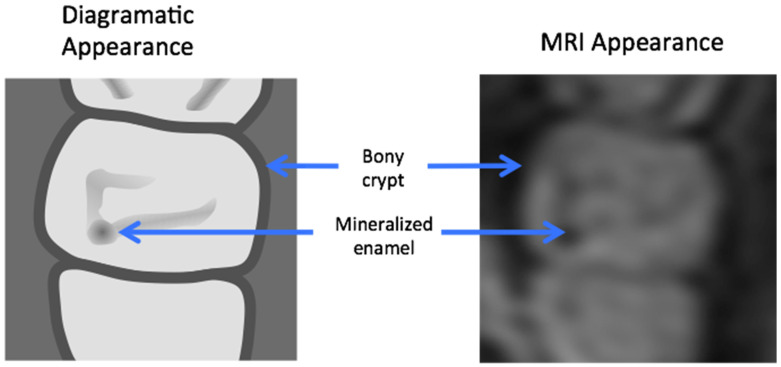
Post-appositional tooth germ anatomy shown diagrammatically (**left**) and as seen on axial MRI (**right**). On MRI, mineralized structures appear hypointense (dark), whereas water/cellular components are hyperintense (bright).

**Figure 18:**
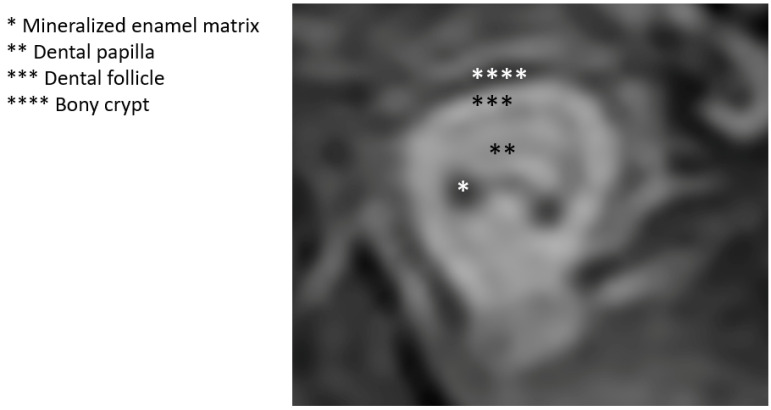
Post-appositional tooth germ anatomy shown on T2-weighted coronal MRI. Note the hypointense (dark) signal corresponding to hypocellular mineralized tissues (bony crypt and enamel).

A closer look at the MRI appearance of a developing tooth germ in a post-appositional stage demonstrates an envelope of partially mineralized, hypo-intense enamel and dentin derived from the enamel organ and outer layer of dental papilla, respectively. This hypointense layer envelops the non-mineralized dental papilla, which demonstrates enhancement and T2 hyperintensity. Surrounding the enamel organ and dental papilla, the dental follicle exhibits similar MR attributes as the dental papilla, as it is composed of the same ectomesenchyme derived from neural crest cells. All tooth germs develop inside a bony crypt. The wall of the crypt is comprised of compact bone, which presents as a hypointense rim surrounding the tooth germ on MRI (Figure 17 and Figure 18).

**Figure 19:**
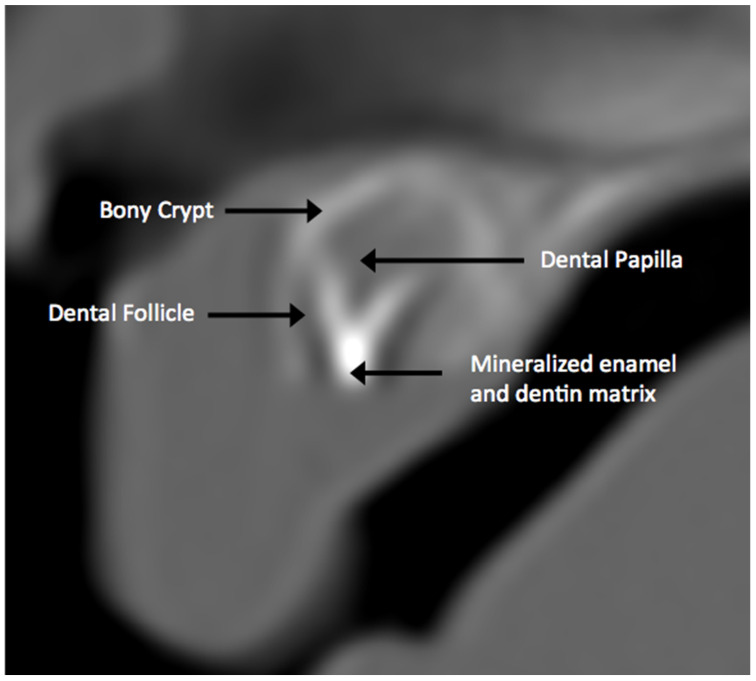
Post-appositional tooth germ anatomy of a central incisor on CT. Note the hyperdense appearance of the mineralized bony crypt and mineralizing crown (enamel and dentin) of the tooth relative to the hypodense appearance of the dental follicle and papilla.

**Figure 20:**
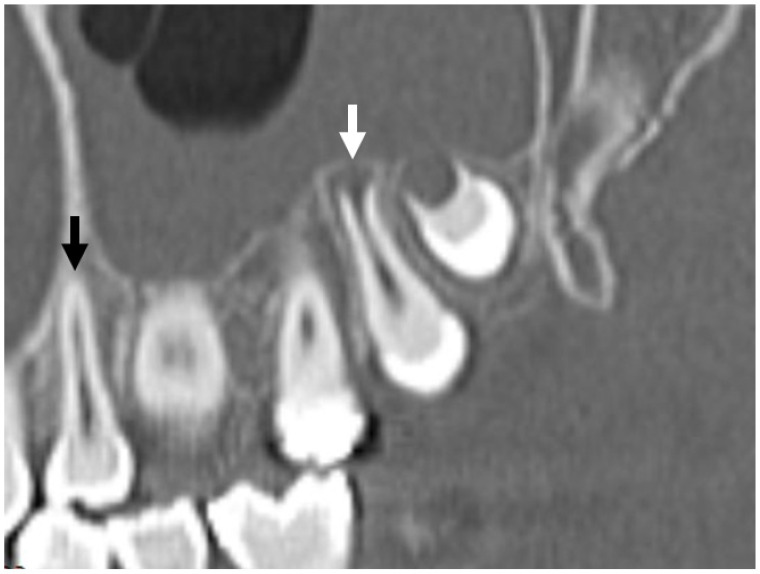
Obliquely reformatted CT demonstrating a mature root apex of the maxillary first premolar (black arrow) and the developing root of maxillary second molar palatal root. Note the flared appearance of the root apex with a “blunderbuss” appearance (white arrow). The increased lucency around the apex should not be mistaken for pathology.

On CT, post-appositional tooth germs demonstrate hyperdensity of the developing crown (enamel and dentin), with relative hypodensities corresponding to the unmineralized dental papilla and dental follicle. The bony crypt housing the tooth germ, being comprised of bone, is hyperdense on CT (Figure 19). Prior to the convergence of the root sheath, the root apex is open and flared with a “blunderbuss” appearance (Figure 20) [38]. Knowledge of this normal appearance can help differentiate root resorption from a pathologic process vs. normal development. Comparison with the contralateral side is also helpful.

**Figure 21:**
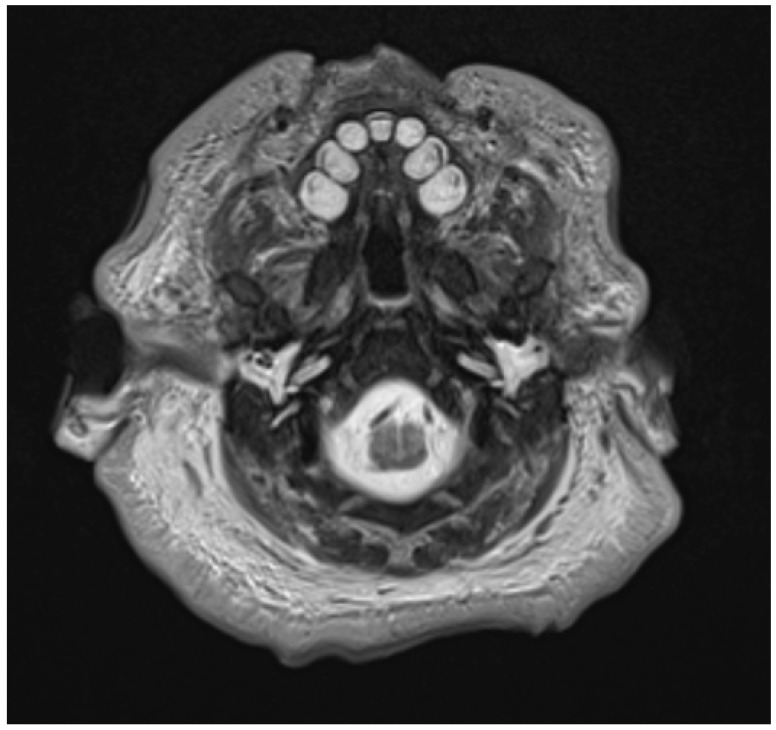
Eight-day old male with holoprosencephaly. Axial T2-weighted MRI demonstrates a single central incisor tooth germ. This appearance is associated with holoprosencephaly the diagnosis and should direct attention to potentially associated abnormalities in the brain.

**Figure 22:**
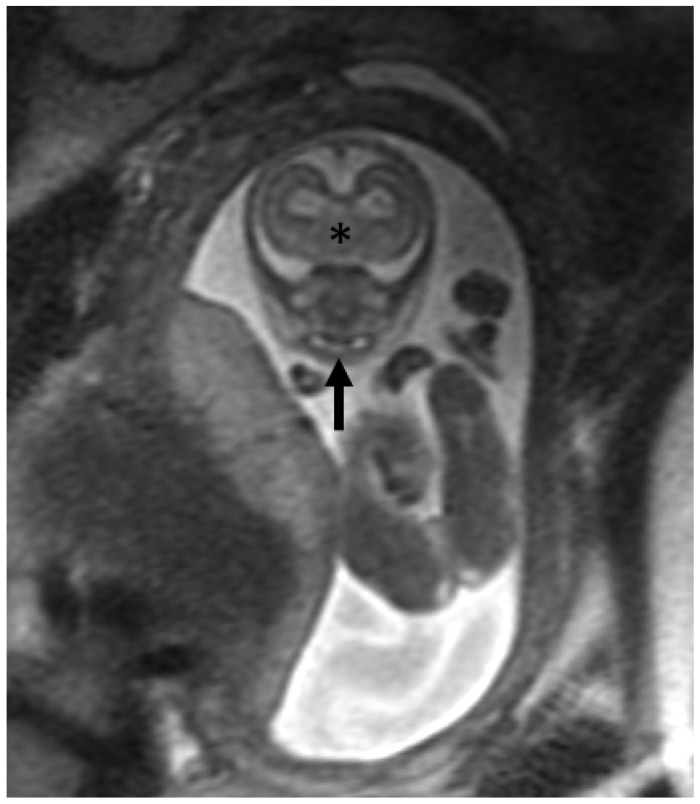
Fetal T2-weighted MRI showing single central incisor of a patient (up arrow) and the failed thalamic separation (*) compatible with holoprosenchephaly.

**Figure 23:**
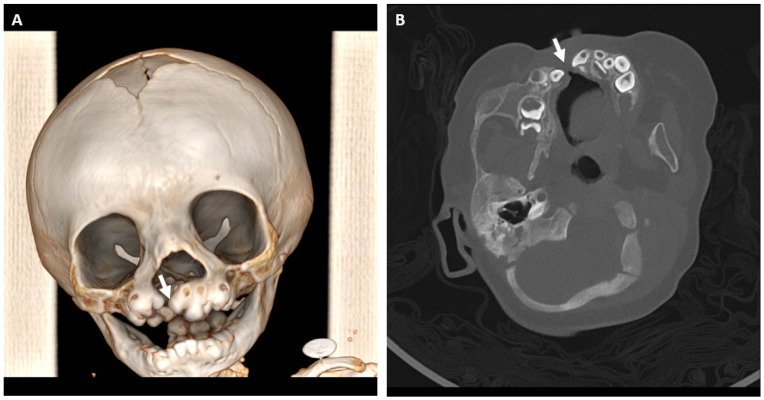
(**A**) Three-dimensional CT reconstruction of a patient with CHARGE syndrome and cleft lip and palate (arrow). (**B**) The presence of a cleft through the dental arch is frequently associated with missing and ectopic teeth (arrow).

**Figure 24:**
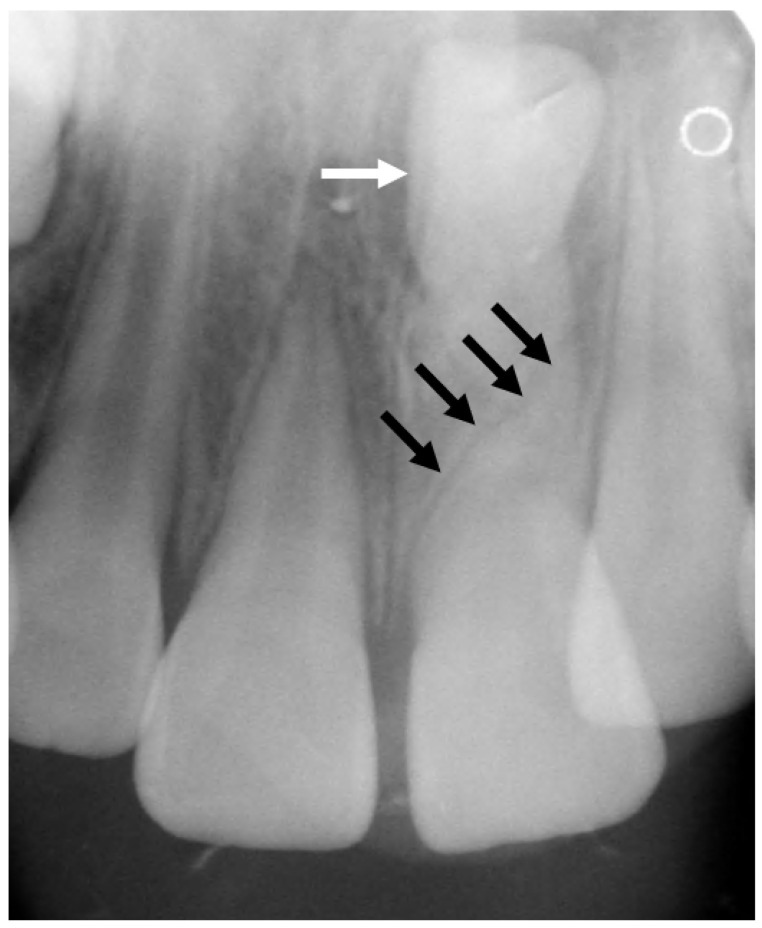
Dedicated dental radiograph demonstrating the presence of an unerupted supernumerary mesiodens (white arrow), which exerted mass effect on the left maxillary central incisor, resulting in a deformation deformity known as a dilacerated root (black arrows).

The etiologies of abnormal dental development are variable and it is often not feasible to determine the etiology radiographically. However, understanding the types of abnormalities can occasionally suggest an underlying cause (Figure 21 and Figure 22). A malformation is an abnormality in morphogenesis secondary to an intrinsically abnormal developmental process. A disruption is an alteration to a previously normal developmental process (Figure 23). A deformation is an abnormality in morphogenesis secondary to abnormal forces on a normal developmental process. This is exemplified by space constrictions placed on a developing root resulting in an abnormal curvature termed a dilacerated root (Figure 24) [39].

### 3.4. Application of Principles to Consecutive MR Exams

The information and principles outlined above were retrospectively applied to a series of fifty patients who underwent neonatal brain magnetic resonance imaging for a variety of reasons. Fifty consecutive-term (≥38 weeks gestation) neonatal patients (mean age 8.7 ± 8.1 days, range 0–27 days, median 6.5 days) who underwent brain MR at our institution were retrospectively analyzed after IRB approval. Review was performed by two neuroradiologists with ABR subspecialty certificates in neuroradiology with clinical practices focusing on pediatric neuroradiology. There were 25 males and 25 females. The most common clinical indications for the MRI examination included seizure (n = 16), hydrocephalus (n = 8), and hypoxic ischemic encephalopathy (n = 7).

Overall, the tooth germs were small, with the maximum size of 4–5 mm in greatest dimension in nearly all patients. All 20 deciduous teeth were visible in every patient. In all 50 patients, the primary teeth were in the early maturation stage of development with little to no root development. Greater mineralization was seen in the crowns (post-appositional phase) of primary first molars when compared to the second molars. Permanent tooth mineralization was not observed in any patient. Except for a single patient, all patients had bilateral pre-appositional-stage permanent mandibular and maxillary central and lateral incisors as well as bilateral maxillary and mandibular permanent first molars developing posterior to the primary second molars. The exception was a child with Down syndrome who had only central maxillary incisors and mandibular molar permanent teeth germs at the time of imaging. In 36 of 50 patients (72%), permanent bilateral maxillary and mandibular canine tooth germs were visible. No premolar, second molar, or third molar tooth germs were visualized.

### 3.5. Anatomical and Imaging Correlations to Odontogenic Infectious Disease

A thorough understanding of dental maturation not only facilitates diagnosis of disorders of development but can also aid in the diagnosis of pathologic processes of fully formed teeth, the most common of which include odontogenic infections. Odontogenic infection can be categorized into diseases of the tooth (decay) or the periodontal supporting structures (periodontal disease). Many clinical and radiological reports on infectious disease involving the dentition and supporting tissues do not distinguish between decay and periodontal disease. In fact, these are two distinct entities with unique etiologies, pathophysiologies, and treatments. Knowledge of the anatomy and common imaging appearances can assist in distinguishing these two sources of odontogenic infection.

#### 3.5.1. Dental Decay Pathophysiology

Despite its colloquial name, dental decay is an infection like any other, with a bacterial pathogen, the requirement of a susceptible host, and a food substrate [40]. The decay process begins with biofilm formation; bacterial consumption of carbohydrates, resulting in acid production; and demineralization of the outermost tooth structure—enamel. This stage is asymptomatic. As the process continues, the next mineralized layer encountered is the dentin, an inorganic hard tissue produced by living cells located in the soft tissue pulp deep to the dentin. These cells send processes out into the dentin, which can sense temperature and osmotic gradients. This stage is often symptomatic, presenting with sensitivity to sweet or cold food or drink. If the disease goes untreated, the decay will progress to involve the pulp causing pulpitis. Patients with pulpitis often have lingering sensitivity to painful stimuli. At this stage, the pain cannot be localized to a specific tooth, as the pulpal tissue has pain but not proprioceptive fibers. When bacteria exit the apex of the root canal, the surrounding periodontal tissues become inflamed; this condition is called periapical periodontitis. This stage of the disease presents with well-localized pain to palpation of the involved tooth, as the periodontal tissues contain proprioceptive nerve fibers, unlike the pulp. Again, if untreated, spread of infection through the bony cortex can result in a subperiosteal or soft tissue abscess formation and cellulitis with or without systemic involvement.

#### 3.5.2. Dental Decay Radiologic Correlations

**Figure 25:**
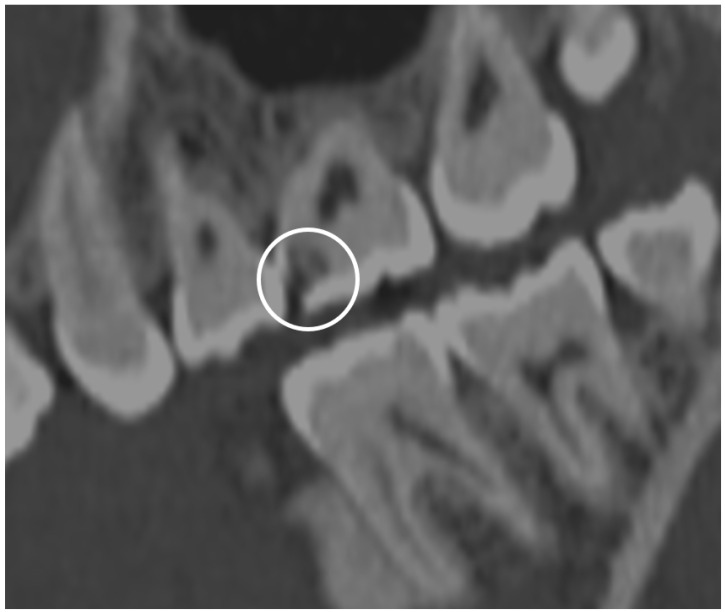
Sagittal section CT demonstrating lucency (white circle) within the enamel and dentin layer of a maxillary first molar, compatible with decay.

While dedicated dental radiographs provide optimal evaluation of the teeth, these are not routinely encountered by radiologists. More likely, a panoramic radiograph or a CT will be obtained. Regardless of the modality, the hallmark image findings of decay are the same—radiolucency of the crown and periapical tissues. During the initial stages of decay resulting in demineralization, the hallmark image finding is lucency of the crown (Figure 25). As demineralization continues, cavitation of the crown may occur, yielding the radiographic appearance of missing coronal tooth structure [6,41].

**Figure 26:**
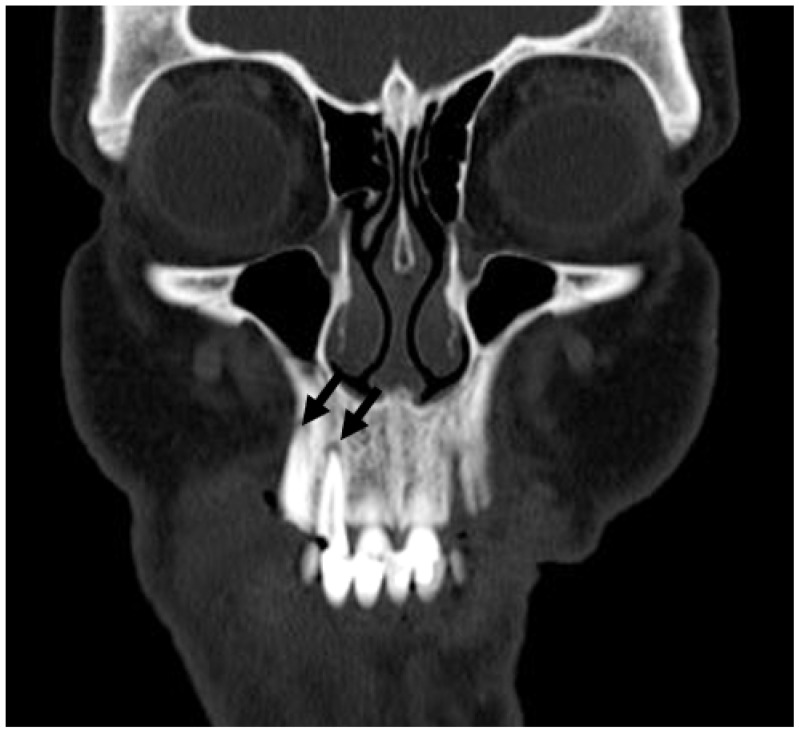
Coronal CT demonstrating periapical radiolucencies (arrows) involving decayed right maxillary lateral incisor and canine, indicative of periapical periodontitis.

Extension of decay into the pulp chamber may occur with or without cavitation resulting in pulpitis. The pulpitis stage cannot be identified radiographically. The progression of bacterial proliferation through the canal and into the periapical periodontal tissues results in a demineralization of the alveolar bone, manifesting as a periapical radiolucency on radiograph or CT (Figure 26) or hyperintensity on T2-weighted MRI [42]. It is important to note that periapical periodontitis can be present without a visible periapical radiolucency [43]. Further, in the case of a tooth treated with root canal therapy, the periapical radiolucency may persist indefinitely even after successful treatment. Importantly, in some cases of periapical infection, the surrounding bone becomes sclerotic in a reactive process called condensing osteitis. In other patients, infection spreads through the outer cortex of the maxilla or mandible and results in a subperiosteal abscess and cellulitis [44].

#### 3.5.3. Periodontal Disease Pathophysiology

Periodontal disease begins with the formation of subgingival plaque (biofilm), which causes gingivitis [45]. The inflamed gingiva creates a deep sulcus (the space between the gum and tooth), which is difficult to clean and is relatively oxygen poor. This local environment is conducive to the proliferation of pathologic bacteria known to cause periodontal disease, specifically, Gram-negative rods, which secrete a lipopolysaccharide endotoxin. This toxin is highly inflammatory and produces a robust host response that leads to the destruction of alveolar bone, periodontal ligament and the gingival attachment to the tooth. This results in an even deeper gingival pocket that is even more difficult to clean. The progressive loss of periodontal support ultimately leads to tooth loss. At any point during this process, an abscess can form within the periodontal tissues, an occurrence that is typically visible clinically.

#### 3.5.4. Periodontal Disease Radiologic Correlations

**Figure 27:**
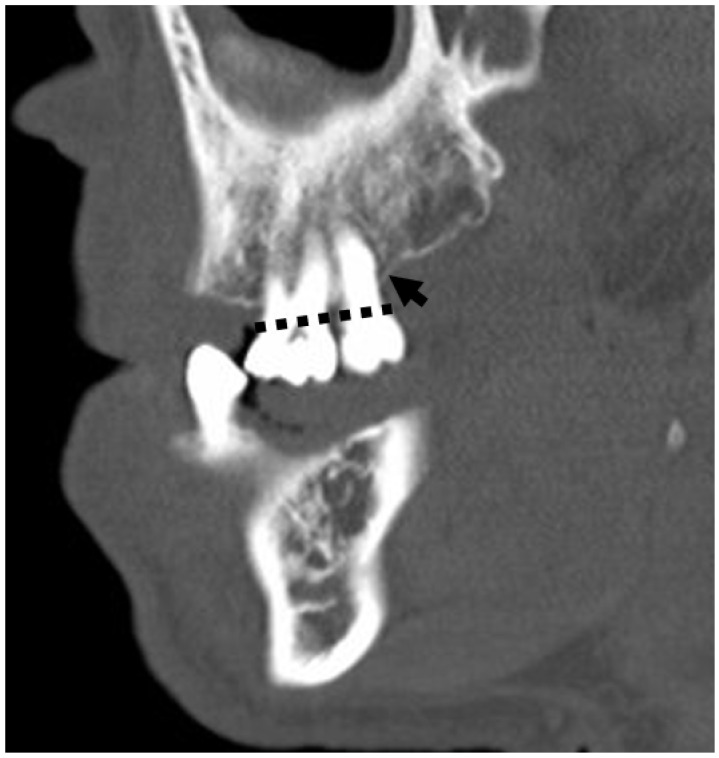
Sagittal section CT demonstrating horizontal bone loss, a sequela of periodontal disease. Note the alveolar crest (arrow) below its normal location at the crown–root junction (dashed line).

**Figure 28:**
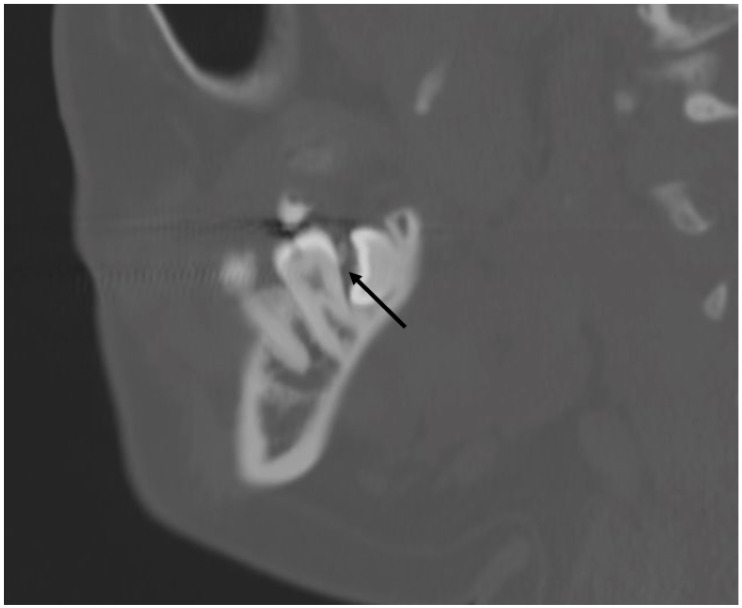
Sagittal section CT showing a vertical bony defect (arrow) posterior to the mandibular second molar, indicative of periodontal disease. In this case, the mesial-angulated third molar is contributing to the vertical bony defect, precluding adequate hygiene.

The hallmark imaging finding of periodontal disease is bone loss. However, unlike in decay, the bone loss begins at the alveolar crest and progresses inferiorly [46]. There are two general categories of bone loss, horizontal and vertical. Figure 27 demonstrates an example of horizontal bone loss. Depending on the state of the adjacent soft tissues, horizontal bone defects may or may not represent active disease but imply that periodontal tissue destruction has occurred either from present or past disease. A vertical bony defect, on the other hand, suggests active disease. This is because a vertical bony defect is almost always accompanied by a deep gingival sulcus, which is unhygienic and very conducive to periodontal pathogenic bacterial proliferation (Figure 28).

## 4. Conclusions

This article reviews normal dental anatomy and development and describes the MRI and CT appearance of developing teeth in the neonatal period. This period is comprised of very active development, with various stages of maturation, resulting in a varied radiologic appearance. Knowledge of normal radiologic appearances of developing and mature teeth can help in the identification of congenital and acquired pathology as well as the prevention of misdiagnosis of normal development as pathologic.

### Future Directions

Despite the inclusion of the dentition on several radiologic examinations obtained for non-dental pathology, little is taught in medical school or radiology residency pertaining to the normal appearance of developing teeth. The dissemination of knowledge in a formalized manner is critical to bridging this knowledge gap. Further, opportunistic screening for odontogenic disorders on examinations ordered for other indications can improve patient outcomes and should be explored.

## Figures and Tables

**Table 1:** Key terms in dental development and embryology.

Ectomesenchyme	Mesenchymal Tissue Derived from Neural Crest Cells (Ectoderm)
Tooth germ	A developing tooth containing enamel organ, dental papilla, and dental follicle. Also referred to as tooth anlagen.
Enamel organ	Epithelial-derived structure that helps form the crown of a developing tooth, specifically providing the cells that produce enamel.
Dental papilla	Condensed mass of ectomesenchyme surrounded by enamel organ, which ultimately forms the dentin and pulp of the developing tooth.
Dental follicle	Ectomesenchyme surrounding dental papilla, which becomes cementum and periodontal ligament. Also referred to as dental sac.

## Data Availability

No new data were created or analyzed in this study. Data sharing is not applicable to this article.
